# Exome sequencing revealed a novel loss‐of‐function variant in the GLI3 transcriptional activator 2 domain underlies nonsyndromic postaxial polydactyly

**DOI:** 10.1002/mgg3.627

**Published:** 2019-05-21

**Authors:** Muhammad Umair, Naveed Wasif, Alia M. Albalawi, Khushnooda Ramzan, Majid Alfadhel, Wasim Ahmad, Sulman Basit

**Affiliations:** ^1^ Medical Genomics Research Department, King Abdullah International Medical Research Center (KAIMRC) King Saud Bin Abdulaziz University for Health Sciences, Ministry of National Guard‐Health Affairs (MNGHA) Riyadh Saudi Arabia; ^2^ Institut für Human Genetik Ulm Universität Ulm Germany; ^3^ Center for Genetics and Inherited Diseases Taibah University Medina Saudi Arabia; ^4^ Department of Genetics King Faisal Specialist Hospital and Research Centre Riyadh Saudi Arabia; ^5^ Division of Genetics, Department of Pediatrics King Abdullah Specialized Children’s Hospital (KASCH) Riyadh Saudi Arabia; ^6^ Department of Biochemistry Quaid‐i‐Azam University Islamabad Pakistan

**Keywords:** GLI3, loss‐of‐function variant, PAPA, polydactyly, Sanger sequencing, whole exome sequencing

## Abstract

**Background:**

Polydactyly is a common genetic limb deformity characterized by the presence of extra fingers or toes. This anomaly may occur in isolation (nonsyndromic) or as part of a syndrome. The disease is broadly divided into preaxial polydactyly (PPD; duplication of thumb), mesoaxial polydactyly (complex polydactyly), and postaxial polydactyly (PAP: duplication of the fifth finger). The extra digits may be present in one or both the limbs. Heterozygous variants in the *GLI3*, *ZRS*/*SHH*, and *PITX1* have been associated with autosomal dominant polydactyly, while homozygous variants in the *ZNF141*, *IQCE*, *GLI1*, and *FAM92A* have been associated with autosomal recessive polydactyly. Pathogenic mutations in the *GLI3* gene (glioma‐associated oncogene family zinc finger 3) have been associated with both nonsyndromic and syndromic polydactyly.

**Methods:**

Here, we report an extended five generation kindred having 12 affected individuals exhibiting nonsyndromic postaxial polydactyly type A condition. Whole‐exome sequencing followed by variant prioritization, bioinformatic studies, Sanger validation, and segregation analysis was performed.

**Results:**

Using exome sequencing in the three affected individuals, we identified a novel heterozygous frameshift variant (c.3567_3568insG; p.Ala1190Glyfs*57) in the transcriptional activator (TA2) domain of the GLI3 encoding gene.

**Conclusion:**

To the best of our knowledge, the present study reports on the first familial case of nonsyndromic postaxial polydactyly due to the *GLI3* variant in Pakistani population. Our study also demonstrated the important role of GLI3 in causing nonsyndromic postaxial polydactyly.

## 1 INTRODUCTION

Polydactyly or hexadactyly is a common congenital limb deformity evident prenatally or instantly after birth. Polydactyly has a general population incidence of approximately 1.6−10.7/1,000 in live births (Malik, 2014; Umair, Ullah, Abbas, et al., 2018). To date, more than 300 syndromic forms of polydactyly have been characterized. Polydactyly is classified into three main types, postaxial polydactyly (PAP; ulnar) where the extra digit is located along fifth digit, preaxial (PPD; radial side) when the extra digit is located along the thumb or great toe, and complex polydacyly or mesoaxial polydactyly, when the extra digit originate between the second, third, and fourth digits (Umair, Ullah, Abbas, et al., 2018).

Postaxial polydactyly is the duplication of little finger. It is categorized into two types: the postaxial type A (PAPA; well‐developed extra digit) and the postaxial type B (PAPB; hypoplastic rudimentary extra digit or a skin tag or appears as a small protuberance). Polydactyly may occur as a nonsyndromic form (isolated) or in association with other severe abnormalities (syndromic form) such as Bardet–Biedl syndrome, split hand/foot malformation, syndactyly, and Ellis–Van Creveld syndrome, respectively (Khan et al., 2012; Ullah, Gul, et al., 2018; Ullah, Khalid, et al., 2018; Ullah, Umair, et al., 2017 Umair, Ahmad, Bilal, & Abbas, 2018; Umair, Ahmad, Bilal, Ahmad, & Alfadhel, 2018; Umair, Seidel, et al., 2017).

Until now, only six genes including *ZNF141* on chromosome 4p16.3, *IQCE* on 7q21‐34, *ZRS*/*SHH* on 7q36, *GLI3* on 7p14.1, *GLI1* on 12q13.3, *FAM92A* on 8q22.1, and three other loci (13q21‐32, 13q13.3‐21.2, and 19p13.1‐13.2) have been associated with nonsyndromic postaxial polydactyly (Palencia‐Campos et al., 2017; Schrauwen et al., 2018; Ullah et al., 2019; Umair, Shah, et al., 2017; Umair, Ullah, Abbas, et al., 2018). Mutations in the *GLI3* gene (MIM 165240) have been associated with five diverse disorders including the Greig cephalopolysyndactyly syndrome (GCPS; MIM 175700), Pallister–Hall syndrome (PHS; MIM 146510), and Acrocallosal syndrome (ACLS; MIM 200990), postaxial polydactyly type A/B (PAP‐A/B; MIM 174200) and preaxial polydactyly type‐IV (PPD‐IV; MIM 174700), and somatic hypothalamic hamartomas (MIM 241800).

In this study, we have ascertained an extended Pakistani family exhibiting typical features of nonsyndromic PAP types A. Whole‐exome sequencing followed by Sanger sequencing revealed a novel frameshift variant in the *GLI3* located on chromosome 7p14.1.

## 2 MATERIALS AND METHODS

### 2.1 Ethical approval and family recruitment

**Figure 1:**
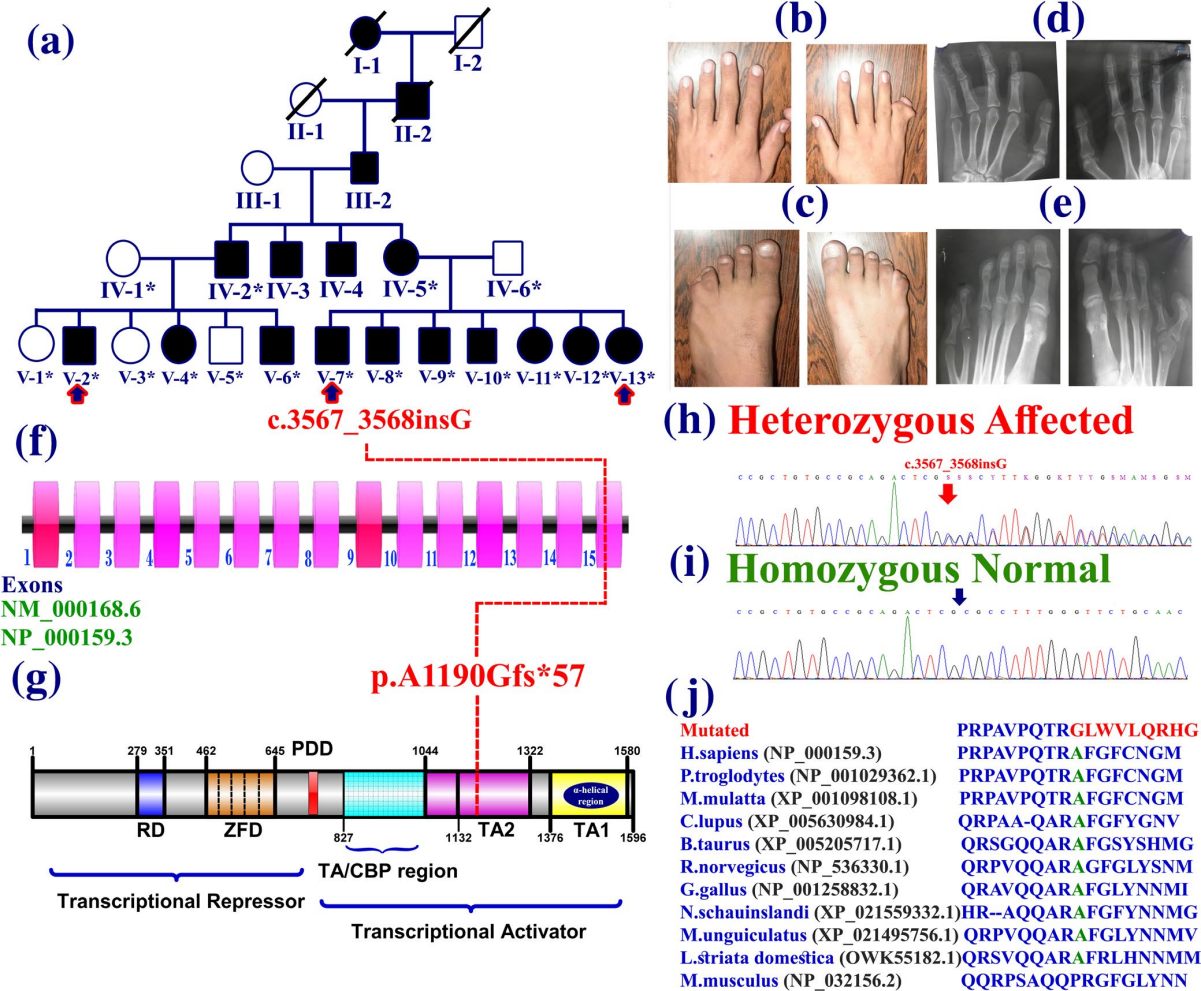
(a) Pedigree of the family showing autosomal dominant pattern of inheritance. Circles and squares represent females and males, respectively. Clear symbols represent normal and filled affected individuals. The individual numbers labeled with asterisks were available for this study. Blue arrow represent those for whom WES was performed (b) Hands of affected individual (V‐2) having fifth–sixth complex synpolydactyly in the right hand and partial syndactly of fourth–fifth figure on left hand. (c) Feet of the affected individual (V‐2) having unilateral fourth–fifth toe syndactly only in left foot. (d, e) Radiographs of the affected individual (V‐7), showing hands after surgical removal of extra digit and feet showing bilateral PAP type A. (f) Schematic representation of 15 exons of the *GLI3* gene. (g) GLI3 protein with different domains and position of identified mutation is shown in red. The intronic regions are not drawn up to scale. (h, i) Sanger sequencing electropherograms of the variant (c.3567_3568insG) identified in the *GLI3* gene. (j) Conservation of the mutated amino acid (Ala1190) across different species

Ethical approval for conducting the present study was obtained from Institutional Review Board of Quaid‐i‐Azam University Islamabad, Pakistan. The family was recruited from the Punjab province of Pakistan. Affected and unaffected individuals of the family were briefed about aims and objectives of the project in local language. All the participating individuals signed the informed consent and approved publication of data, photographs, and radiographs. A total of 16 affected individuals including six females and 10 males were observed in five generations with no generation skipping exhibiting autosomal dominant inheritance pattern (Figure 1a).

### 2.2 Blood collection and extraction of genomic DNA

Pedigree was drawn according to the standard instructions and detailed interview with family elders clearly depicting autosomal dominant inheritance. Blood samples were collected from thirteen affected and five normal individuals (represented by asterisks; Figure 1a) in the EDTA containing vacutainer sets (BD, Franklin Lakes, New Jersey). Genomic DNA was extracted from blood samples using commercially available kit following standard protocols.

### 2.3 Whole‐exome sequencing

DNA of three affected individual (V‐2, V‐7, V‐13) was exome sequenced using Illumina HiSeq 2500 (Illumina, San Diego, CA) and libraries were prepared using the Agilent SureSelect Target Enrichment Kit as described earlier (Umair, Ullah, Abbas, et al., 2018). Owing to the dominant inheritance pattern observed in the pedigree (Figure 1a), only heterozygous variants were filtered and further validated using Sanger sequencing.

### 2.4 Sanger sequencing

The *GLI3* sequence was downloaded from Ensembl Genome Browser (http://www.ensembl.org/). Specific primers for the identified variant were designed using “Exon Primer” (http://ihg.gsf.de). Sanger sequencing was performed using standard methods as described earlier (Umair et al., 2016).

### 2.5 Insilco analysis

Pathogenicity of the identified variants was calculated using MutationTaster (http://www.mutationtaster.org/), VarSome (https://varsome.com/), Polymorphism Phenotyping V2 (PolyPhen‐2) (http://genetics.bwh.harvard.edu/pph2/), PredictProtein (https://predictprotein.org/), and Sorting Intolerant From Tolerant (SIFT) (http://sift.bii.a-star.edu.sg/). Amino acid [Ala1190] conservation in the different *GLI3 *orthologs was scrutinized using NCBI homologene (http://www.ncbi.nlm.gov/homologene/).

## 3 RESULTS

### 3.1 Clinical description

The postaxial polydactyly type A (PAPA) was observed as the hallmark feature in all affected individuals, while most of the affected individuals had undergone surgical procedure. The PAP type A was observed in both hands and feet, mostly affecting all four autopods (13/16). Affected individuals presented PAP with well‐developed extra figure, while syndactyly of the fourth and fifth digits/toe and polysyndactyly of fifth–sixth figures/digits was also observed in most of the affected individuals (9/16) (Figure 1b,c). There were no neurological, craniofacial, cardiovascular, obesity, ophthalmological abnormalities observed in the affected and normal individuals of the family. The extra digits were nonfunctional and presented fixed flexion deformity and caused difficulty in daily life, thus surgically removed in most of the cases (Figure 1d). Radiographical examination of the hands of affected individuals showed normal presentation after surgery while the feet showed duplication at metatarsal level in the left foot, while duplication in right foot occurs at the fifth digit, giving a two headed appearance (Figure 1e). Features such as height, limb length, head shape and circumference, facial dysmorphism, throat (epiglottis), and deafness (audiogram; pure‐tone hearing test), were also examined in order to rule out different syndromic defects.

### 3.2 WES and Sanger sequencing

DNA of three affected individual (V‐2, V‐7, V‐13) was subjected to exome sequencing using Illumina HiSeq 2500 (Umair, Ullah, Abbas, et al., 2018). After WES, filters were applied for screening different variants. As the pedigree demonstrated autosomal dominant inheritance pattern, thus only heterozygous variants were given priority. We obtained 61 common heterozygous variants in the exome data of all three affected individuals. Further filtration identified a novel frameshift variant (c.3567_3568insG; p.Ala1190Glyfs*57) in exon 15 of the *GLI3*. Using Sanger sequencing approach, we have demonstrated that the variant is perfectly segregating with the disease phenotype in all members of the family. The mutation (p.Ala1190Glyfs*57) is present in a highly conserved transcriptional activation (TA2) domain (Figure 1j). The identified variant was not observed in the ExAC browser (http://exac.broadinstitute.org/), gnomAD (http://gnomad.broadinstitute.org/), 1,000 Genomes, Pakistan Genetic Mutation database (Qasim et al., 2018) and in 135 in‐house exomes (Pakistani exomes).

## 4 DISCUSSION

Pathogenic sequence variants in the *GLI3* have been associated with both syndromic and nonsyndromic polydactyly. To date, 236 different mutations in the *GLI3* gene has been listed. This included only eight variants causing nonsyndromic polydactyly phenotype. Most of the mutations in the *GLI3* result in syndromic forms including GCPS and PHS.

Affected individuals in the family, presented here, showed nonsyndromic PAP type A phenotype. In few individuals, fourth–fifth figure syndactyly (SDIII) and polysyndactyly of fourth–fifth (SDI‐d) and fifth–sixth toes were observed. Some of the features reported previously to be associated with nonsyndromic polydactyly have not been observed in affected members of our family. This included PAP type B, camptodactyly, zygodactyly, hypoplasticity of the third toe (Mumtaz, Yıldız, Lal, Tolun, & Malik, 2017), hypertelorism, macrocephaly, developmental delay, corpus callosum agenesis/hypoplasia, and motor delay (Démurger & Angers, 2015; Xiang, Wang, Bian, Xu, & Fu, 2016).

Here, using WES and Sanger sequencing, we have identified a novel frameshift variant (c.3567_3568insG) in exon 15 of the *GLI3* (NM_000168.6). The variant (c.3567_3568insG) successfully co‐segregated with the polydactyly phenotype in the family. The identified variant resulted in a frameshift and created a premature stop codon 57 amino acids downstream of the site of mutation (p.Ala1190Glyfs*57). It is highly likely that this mutation might result either in truncated GLI3 protein or complete loss of transcript through nonsense mediated mRNA decay.

The GLI3 functions as an important tissue patterning and developmental regulator. It is one of the three GLI transcription factors (GLI1, GLI2, GLI3) playing important role in the canonical Hedgehog (HH) signaling pathway (Hui & Angers, 2011). Human *GLI3 *is constructed with 15 exons encoding 1,580 amino acids protein. The GLI3 functional domains comprises an N‐terminal transcriptional repressor, five zinc finger (mediate DNA binding), protease cleavage site, CBP‐binding regions (TA/CBP), two C‐terminal transcriptional activation (TA2 and TA1) and an α‐helical region (Figure 1g). The CBP‐binding region expressed ubiquitously and functions as transcriptional co‐activator. The α‐helical acts as an activation domain (Naruse, Ueta, Sumino, Ogawa, & Ishikiriyama, 2010). The mutation identified in the present study is located in the conserved transcriptional activation 2 (TA2) domain, which is predicted to result in loss of the TA1 and the α‐helical region thus resulting in a shorter GLI3 protein.

Several studies have elucidated genotype–phenotype correlation in terms of location of the mutation in particular domain and the resulting phenotype (Ni et al., 2018; Wang et al., 2014). Currently, most *GLI3* mutations causing syndromic and nonsyndromic phenotypes are loss‐of‐function variants (Ni et al., 2018). Mutations in N‐terminal and the C‐terminal regions are mostly associated with the GCPS, while the PHS phenotype mostly results due to mutations in the central part of the protein (Demurger et al., 2015; Jamsheer et al., 2012). The *GLI3* mutation causing isolated polydactyly is not restricted to one specific domain. Our data also supports Wang et al. (2014) observation, that *GLI3* mutations causing nonsyndromic polydactyly are located in all functional domains except the TA/CBP domain. Identification of more nonsyndromic polydactyly cases due to *GLI3* mutations will help to further outline such association and present proper genotype‐phenotype correlations.

In conclusion, we have reported first study of a novel loss‐of‐function variant in the *GLI3* responsible for nonsyndromic PAP type A in a Pakistani family. The present study increase the mutation spectrum of *GLI3* associated pathogenesis and also addresses a thought whether a specific mutation type is a separate identity, which might lead to different digit/limb deformities.

## ETHICS STATEMENT

Written informed consent for the publication of the data, photographs, and radiographs was obtained from all the participating members.

## CONFLICT OF INTERESTS

None declared.
